# Classification and Automated Interpretation of Spinal Posture Data Using a Pathology-Independent Classifier and Explainable Artificial Intelligence (XAI)

**DOI:** 10.3390/s21186323

**Published:** 2021-09-21

**Authors:** Carlo Dindorf, Jürgen Konradi, Claudia Wolf, Bertram Taetz, Gabriele Bleser, Janine Huthwelker, Friederike Werthmann, Eva Bartaguiz, Johanna Kniepert, Philipp Drees, Ulrich Betz, Michael Fröhlich

**Affiliations:** 1 Department of Sports Science, Technische Universität Kaiserslautern, 67663 Kaiserslautern, Germany; eva.bartaguiz@sowi.uni-kl.de (E.B.); michael.froehlich@sowi.uni-kl.de (M.F.); 2 Institute of Physical Therapy, Prevention and Rehabilitation, University Medical Centre, Johannes Gutenberg University Mainz, 55122 Mainz, Germany; juergen.konradi@unimedizin-mainz.de (J.K.); claudia.wolf@unimedizin-mainz.de (C.W.); janine.huthwelker@unimedizin-mainz.de (J.H.); ulrich.betz@unimedizin-mainz.de (U.B.); 3 Department Augmented Vision, German Research Center for Artificial Intelligence, 67663 Kaiserslautern, Germany; bertram.taetz@dfki.de (B.T.); gabriele.bleser@dfki.de (G.B.); 4 Department of Orthopedics and Trauma Surgery, University Medical Centre, Johannes Gutenberg University Mainz, 55122 Mainz, Germany; fwerthma@students.uni-mainz.de (F.W.); johanna.kniepert@unimedizin-mainz.de (J.K.); philipp.drees@unimedizin-mainz.de (P.D.)

**Keywords:** spine, machine learning, artificial intelligence, data mining, biomechanics, back pain, osteoarthritis, spinal fusion, explainable artificial intelligence

## Abstract

Clinical classification models are mostly pathology-dependent and, thus, are only able to detect pathologies they have been trained for. Research is needed regarding pathology-independent classifiers and their interpretation. Hence, our aim is to develop a pathology-independent classifier that provides prediction probabilities and explanations of the classification decisions. Spinal posture data of healthy subjects and various pathologies (back pain, spinal fusion, osteoarthritis), as well as synthetic data, were used for modeling. A one-class support vector machine was used as a pathology-independent classifier. The outputs were transformed into a probability distribution according to Platt’s method. Interpretation was performed using the explainable artificial intelligence tool Local Interpretable Model-Agnostic Explanations. The results were compared with those obtained by commonly used binary classification approaches. The best classification results were obtained for subjects with a spinal fusion. Subjects with back pain were especially challenging to distinguish from the healthy reference group. The proposed method proved useful for the interpretation of the predictions. No clear inferiority of the proposed approach compared to commonly used binary classifiers was demonstrated. The application of dynamic spinal data seems important for future works. The proposed approach could be useful to provide an objective orientation and to individually adapt and monitor therapy measures pre- and post-operatively.

## 1. Introduction

In outpatient care, back pain, and knee joint osteoarthritis are among the 30 most common individual diagnoses, with approximately 20% of 18–79-year-olds having physician-diagnosed osteoarthritis, whereby knee and hip joints are most commonly affected [1]. Back pain is considerably lowering the quality of life across all income and age groups and is now the leading cause of disability worldwide [2] with a point prevalence of 25–40% [3], a 12-month prevalence of approximately 61% [4], for example in the German population, and a lifetime prevalence in the American population of up to 85% [5]. Hence, more emphasis should be put on the back. Back pain may be due to a specific vertebrogenic cause, such as spondylitis, herniated disc, or spinal stenosis [6]. However, 90% of back pain is stated to be nonspecific, whereby no anatomic correlate can be detected as the specific cause that requires treatment [7]. Non-specific back pain can be caused by altered function; moreover, pathologies, such as knee or hip osteoarthritis, may cause postural changes or back pain. This is because the body reacts to a pain stimulus with avoidance in both stance and gait, which leads to poor or relieved postures [8]. For those patients diagnosed with a specific cause of their back pain, such as spondylolisthesis, surgery is often required. In the field of spondylodesis, in particular, there has been a large increase in surgery rates in recent years [9,10], even though patients do not always clearly benefit from this type of surgery—most notably, those with persisting pain [11,12]. Hence, objectifiable data, such as radiography or magnetic resonance imaging, are not able to explain this dissatisfying postoperative outcome alone. Besides daily-life monitoring data of wearables [13,14], a posture and motion analysis, combined with artificial intelligence, might be able to provide useful insights.

Artificial intelligence (AI) and machine learning approaches are gaining increasing interest in the field of biomechanical clinical data analysis and have obtained promising results (e.g., after stroke [15] or in Parkinson´s disease [16]). They have proven to be useful in analyzing complex and multivariate data, giving objective orientation and finding discriminative group-specific differences [17,18]. Furthermore, they have even shown advantages over commonly used inference-based statistical analysis methods in those databases [19,20,21]. In the clinical context, they are able to identify pathologic characteristics and even surpass human guidance in the detection of diseases [22,23]. Additionally, they might be able to reduce false-positive mistakes and differences in disease detection based on the different experience levels of the medical practitioners [24]. However, regarding the application of machine learning methods on spinal data for the mentioned pathologies, there is a lack of research. Using machine learning approaches, existing classification studies have investigated pathologies such as osteoarthritis [25] or total hip arthroplasty [17,26]. To the best of the authors’ knowledge, no studies regarding back pain and spinal fusion (spondylodesis) are currently available.

Many machine learning models often show black box characteristics and a lack of transparency [27]. For the user, it is, therefore, hard to trust in the model and its decision because it is opaque—why does the model make certain decisions and what has the model really learned [28]? This opacity does not comply with the requirements of the European General Data Protection Regulation (GDPR, EU 2016/679) [29] and strongly limits practical applications in clinical contexts [30]. Recently, through advances in the application of explainable artificial intelligence (XAI) methods in the biomechanical clinical domain, machine learning is becoming more and more applicable in practical clinical settings [31,32]. XAI offers methods for increasing the trustworthiness and transparency of black box models [27]. The prominent interpretation tools are, for example, Local Interpretable Model-Agnostic Explanations (LIME) [33], SHapley Additive exPlanations (SHAP) [34], and Deep Learning Important FeaTures (DeepLIFT) [35]. The usage of XAI has shown to be particularly useful in understanding individual pathologic differences and is, therefore, of high relevance in the context of personalized medicine, e.g., to monitor therapy measures pre- and post-operatively [31,32]. 

Most works are based on binary classification tasks for knowledge discovery through distinguishing between healthy subjects and subjects with a certain pathology (e.g., [17,25,26,31,32]). For model training, both the data of healthy subjects and of the pathology of interest are often used. The resulting models are, therefore, only applicable for a certain pathology—they are pathology-dependent; hence, they are only able to detect the certain pathology for which they have been trained. To obtain a model for another pathology of interest, completely new training with subjects of the respective pathology has to be performed, even if the training data of healthy subjects remain the same.

Differences in comparison to a healthy group are usually of interest in the clinical context. Therefore, the question arises if it is possible to develop a pathology-independent classifier by only learning the characteristics of healthy subjects in order to recognize any (pathological) deviations. Unsupervised outlier detection methods may have the potential to perform this. A previous work [36] used a one-class support vector machine (OCSVM) and obtained the first promising results. Transferred to the terminology of an outlier detection task, a decision function was learned based on regular observations (healthy subjects) and outliers (subjects with a pathology; in the quoted case, total hip arthroplasty, and one transfemoral amputee) detected due to their location outside the learned frontier.

Overall, there is a lack of research regarding those classifiers and their interpretation. According to current practice, they are treated as black boxes. This does not comply with GDPR and strongly limits applications in the clinical context. Furthermore, it is questionable whether XAI tools are suitable for interpretation in this context. Therefore, the aim of this research is to design and evaluate a pathology-independent classifier that provides explanations of classification decisions using XAI. Using clinical spinal posture data of the clinically highly relevant pathologies back pain, spinal fusion, and osteoarthritis, we wanted to check whether it is possible to adequately explain pathologic differences compared to healthy controls with a pathology-independent classifier and to generate clinically relevant insights. The classification results are compared with those obtained using commonly used binary classification approaches.

## 2. Materials and Methods

### 2.1. Measuring Method, Data, and Subjects

Across four studies, we collected data of 151 subjects. Depending on the study design of the respective study (see Table 1), for every subject, on one or on three separate days, 12 postural data measurements of the spine averaging from 12 to 14 individual images were obtained for each day (36 total measurements for each healthy subject which were recorded on three days). Data were collected of healthy subjects, as well as subjects with various pathologies (back pain, spinal fusion, and osteoarthritis), using the DIERS formetric III 4D™, DICAM v3.7Beta analyzing system as a means for rasterstereography, also called surface topography (ST). All subjects received the same marker placement (vertebra prominens, both dimples, and shoulders). The subjects’ characteristics are described in Table 1. The method allowed to measure the spine in all body planes without the use of invasive or radiation-based approaches or extensive preparation. Recently, in addition to static measurements, this method has proven useful in measuring dynamic spinal data [37,38]. (1 The dataset is part of the dissertation project of Friederike Werthmann; 2 The dataset is part of the dissertation project of Claudia Wolf).

Fifty-five static parameters provided by the system were used (pelvic obliquity (°), pelvic torsion (dimples) (°), pelvic inclination (dimples) (°), pelvic rotation (°), and orientation of VP, T1–12, and L1–L4 in all planes (°)) for modeling. For a detailed description of the parameters, see [37,39] and Appendix A. Data of healthy subjects were used for training the OCSVM. Consequently, only the outliers in the group of healthy subjects influenced the learning of a decision function. Therefore, outliers were removed for the data of the healthy subjects using isolation forest [40] that also pay attention to multivariate outliers. Of the 900 samples, 150 were removed (one subject was completely removed).

For evaluation of the classification performance in terms of the dependence of the class separation, synthetic subject data (*n* = 24 subjects) of known separation to the healthy reference subjects were created. The use of the synthetic data is intended to ensure that groups of different class separations are present to check the performance of the proposed approach in settings with well separated groups as well as settings with little separated groups. To maintain similar spinal characteristics compared to real-world data for the synthetic posture data, data creation was based on the principal components (PCs) of the healthy subjects. After removing the mean and scaling to unit variance, principal component analysis (PCA) was applied. Four different synthetic classes were created, each of which was based on a modification of one PC. Therefore, separately for the first four PCs, data were created by adding random samples from a normal Gaussian distribution. The center of the distribution was set according to the desired cluster separation (silhouette scores of synthetic data compared to healthy data—class S1: 0.49; class S2: 0.41; class S3: 0.33; class S4: 0.16) with a standard deviation equal to 1. Afterward, the synthetic PCA data were transformed back into the original space.

For every real and synthetic subject, 10 samples without replacement were drawn for further calculations.

### 2.2. Data Preprocessing and Model Evaluation

For both the pathology-independent classifier, as well as the binary classifiers, grouped K-fold cross-validation (KFold) with five folds (25–29 healthy subjects were used each time in the training, while 6–9 healthy subjects were used each time in the test set) was used for model evaluation to check if the model was able to generalize to new subjects. Therefore, the data were split considering the subjects to ensure that the samples of each subject were not present in both the training and the testing data. The data of each training fold were split into data for actual training and data for validation (validation size = 20%) to allow hyperparameter searching and probability calibration/calculation without introducing a bias. Standardization was performed based on each respective training set by removing the mean and scaling to unit variance.

### 2.3. Pathology-Independent and Binary Classifier

OCSVM [41] was used for building a pathology-independent classifier. Initially, the SVM algorithm was developed for binary classification tasks. In the case of one-class classification, the algorithm tries to capture the density of the majority class. Outliers are detected as extremes of the learned density function. The OCSVM was trained using training samples of healthy subjects in the training set only. A random hyperparameter search was performed with the parameter “kernel” (linear, radial basis function), “nu,” and “gamma” using the samples of healthy subjects in the validation set for performance evaluation. The remaining hyperparameters were set to the default parameters of Scikit-learn [42]. Validation set performance was measured as the ratio of misclassified healthy subjects to the total number of healthy subjects in the validation set.

The classification output was transformed into a probability distribution over the classes according to Platt´s method [43]. Therefore, a logistic regression model was fitted to the output scores of the model with respect to the actual class labels. In the present case, logistic regression using five-fold CV for hyperparameter selection was fitted to the validation set for each group of subjects.

### 2.4. Validation

For comparing the results of the proposed methodology with those obtained using a classical binary classification method, the random forest classifier (RF) [44] was used (a preliminary study using spinal posture data of another group of subjects showed that the algorithm yielded similar or slightly better classification results compared to other regarded classifiers). A random hyperparameter search was performed with the parameters “n_estimators” and “max_depth” using the validation set performance. The remaining hyperparameters were set to the default parameters of Scikit-learn. The F1 score was used to measure the validation set performance. Probabilities for the RF model were calibrated according to Platt´s method described above.

### 2.5. XAI Interpretations

The model-agnostic interpretation tool Local Interpretable Model-Agnostic Explanations (LIME) [33] was used for explaining the predictions of the OCSVM or the binary classifier using probabilities calculated/calibrated with the described Platt´s method. To explain how a black box model makes a single prediction, LIME performs an approximation of a local prediction of a black box model with a simpler interpretable model. Therefore, data points around an instance of interest of a black box model were generated through perturbation. Afterward, these data points were predicted with the black box model and weighted by their proximity to the selected instance. Finally, an interpretable model was learned on the weighted data points and used for explaining the prediction [33]. Each trained model during KFold was used to explain the predictions of the respective test set. Detailed analyses of the XAI results were performed for the pathology with the highest classification performance. Subject-specific results were exemplarily presented and discussed.

### 2.6. Evaluation Metrics and Calculations

Uncertainties were analyzed using classification probabilities. Due to partly imbalanced classes, predicted probabilities were evaluated using the Brier Skill Score (BSS; normalized by the naive score [45]). Classification performance was reported using the Matthews correlation coefficient (MCC), the F1 score, and a confusion matrix (CM) based on the classification founded on prediction probabilities. Calculations were performed in Python (Python Software Foundation, Wilmington, DE, USA) using Scikit-learn [42] and LIME [33].

## 3. Results

The results for the OCSVM, as well as the binary classification approach, are presented in Table 2. For the pathologies, the best classification performance was obtained for the spinal fusion data. The proposed OCSVM showed improved performance compared to the binary RF classifier for the respective data and the F1 and MCC scores. The BSS was slightly reduced compared to the RF classifier. The worst classification performance was obtained for both approaches for the back pain data. Performance for the osteoarthritis data was between the classifications of spinal fusion and back pain.

For the synthetic data, the binary classifier performed better than the OCSVM (except S2). The difference was especially visible for the data with the lowest separation from the group of healthy subjects (MCC OCSVM: 0.65 ± 0.19; MCC RF: 0.82 ± 0.06). Surprisingly, OCSVM performance was reduced for S1 compared to S2, and even the class separation measured by means of the silhouette score increased for S1.

**Figure 1:**
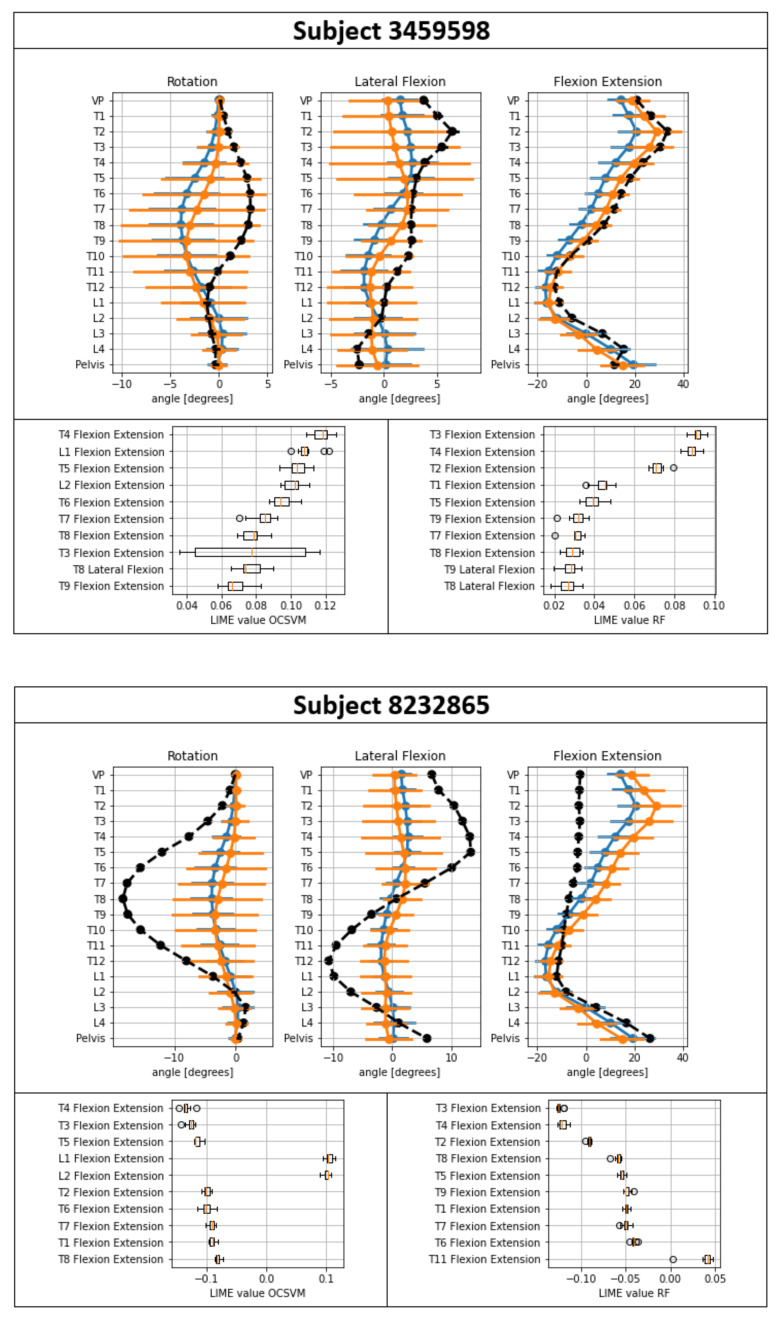
Exemplary posture of one correctly (3459598) and one falsely (8232865) classified subject. Bottom: Displayed LIME values show the effect for the 10 most important features. Negative values represent an effect toward the group of healthy subjects, with positive values indicating an effect that indicates an outlier (patient). Top: Vertebral body positions in the transversal (rotation), coronal (lateral flexion), and sagittal (flexion extension) planes. Positive values indicate a rotation/tilt to the left or ventral (toward flexion), while negative values indicate a rotation/tilt to the right or dorsal (toward extension). Blue = mean and standard deviation (SD) of healthy reference group; orange = mean and SD of group of subjects with the respective pathology; black = mean and SD of the 10 measurements of the subjects of interest.

The local results for the pathology with the highest classification performance (subjects with spinal fusion) for the OCSVM, as well as the binary classifier, are presented in Table 3. Examples for one correctly and one falsely classified subject are given in Figure 1.

Using the mean probability values of the measurements of each subject, 27 and 7 subjects were correctly and falsely classified, respectively, out of the 34 subjects with spinal fusion. For 8 of the 27 correctly classified subjects, the class probability difference was below 0.2. Using the RF approach, 9 and 25 subjects were falsely and correctly classified, respectively. For the majority of the misclassified subjects, the results for the OCSVM and the binary classification approach were congruent.

The point plots in Figure 1 showed, in general, the presence of highly overlapping areas between the classes. Subject 3459598 was correctly classified for both OCSVM and RF with a high probability (probability for classification as an outlier—OCSVM: 1.00; RF: 0.93). For subject 8232865, pathologic differences were clearly visible in the point plot. However, both approaches failed to classify the subject correctly (probability for classification as an outlier—OCSVM: 0.00; RF: 0.37). Looking at the LIME interpretations for both classifiers showed similar features in the top 10 listings. For the correctly classified subject, the LIME values of the top 10 features showed an effect that indicated an outlier. For the misclassified subject, the majority of LIME values for the top 10 features showed an effect toward the class of healthy subjects.

## 4. Discussion

For the discussion of validity/plausibility of the found effects, as well as the proposed pathology-independent classifier, five aspects were addressed in the following: (a) classification performance of the pathology-independent and binary classification approaches, (b) previous research results and biomechanical characteristics in relation to classification performance, (c) LIME effects between the pathology-independent and binary classification approaches, (d) LIME effects in relation to the location of the spinal fusion, and (e) expert knowledge-based interpretation of the exemplary subject results.

(a) Looking at the real data, the best performance was achieved using the pathology-independent classifier for the spinal fusion data (MCC = 0.57 ± 0.23). These results also matched the use of the binary classifier using the RF algorithm trained on both classes (MCC = 0.45 ± 0.25). OCSVM performed better using the spinal fusion data. Therefore, the current study indicated prevalent spinal differences between healthy subjects and subjects after spinal fusion that were suitable for classification. The classification performance of the subjects with osteoarthritis might have indicated potential spinal differences with a limited ability for classification. For the osteoarthritis data, the predicted and actual classes were only weakly correlated when using the stance data (OCSVM: MCC = 0.21 ± 0.12; RF: 0.19 ± 0.21). The results indicated that subjects with osteoarthritis seemed to stand differently compared to healthy subjects. However, the differences seemed relatively small, which resulted in a low discriminative power of the features. An interpretation of the results should, therefore, be carried out with caution. For the back pain data, no good model solution was found for either approach (OCSVM: MCC = 0.13 ± 0.19; RF: 0.08 ± 0.34).

The BSS as a probability scoring metric also quantified the poor distinguishability of subjects with back pain from healthy subjects. Due to the poor results for both the pathology-independent, as well as the binary classifier, it can be ruled out that the proposed approach alone was the cause of the poor results. This was in line with a previous work that demonstrated that even the use of different complex classifiers and metric learning approaches is not able to lead to a significant improvement in the classification performance for subjects with back pain [46].

For the synthetic data, the binary classification approach seemed to show superior performance when the class separation was reduced. This may highlight the importance of highly discriminant data for optimal performance of the pathology-independent approach. Surprisingly, the OCSVM approach showed a lower performance on S1 compared to S2, which was contrary to this statement. It cannot be fully clarified at this point whether this was a random event and, consequently, if further research is necessary.

There were diverging indications that, on the one hand, suggested no clear, systematic performance difference and, on the other hand, that binary classifiers performed better when the class separation was low. This study was intended as an initial investigation, but in order to clarify the mentioned aspects, further research is necessary. However, overall, the results seemed relatively congruent for the OCSVM, as well as for the binary RF approach.

(b) It can be assumed that non-specific back pain and osteoarthritis affect the spine more in terms of dynamic function than in terms of posture. Therefore, these pathologies are less detectable in static measurements. Back pain often originates in the muscular system [47]. Due to an altered movement sequence, other muscle activities, angular courses, and greater joint moments are conceivable and probable [7]. Muscular imbalances are also easier to recognize in dynamic function. Osteoarthritis patients, on the other hand, have postural changes due to contractures in the affected joint, which should also be recognizable in static measurements of the cranially located vertebrae [48,49], which would explain why their detection was more successful. However, the entire spine was demonstrated to compensate for the flexed hip joint in the sagittal plane, but since this is limited, a strongly unbalanced spine–pelvis alignment was found [48]. This compensatory phenomenon should actually be identifiable by the proposed methodology. Nevertheless, since our individual vertebral body positions did not necessarily reflect the spinal position as a whole, our classification approach might appear as not sensitive enough. In spondylodesis patients, the underlying pathologies were directly in the measurement area. As a result, the vertebral bodies to be measured usually no longer contribute to a physiological position, since the affected segments were fixed. After spondylodesis surgery, structural alterations such as a slight shortening of the spine have been found [50], but only after one year, and none of them in the frontal plane. The authors hypothesized that these measurable changes were due to other structural changes, such as muscle shortening and deformed vertebrae. Years of pain with functional alterations of movement patterns and the resulting pathological posture, thus, appeared to have a major lasting impact on anatomical structure [51]. In addition, it can be assumed that neighboring segments compensated for the stiffened area, which is why stronger vertebral body rotations could be recognizable here. Existing surgical scars, as well as spinal hardware such as rods and screws, could also have an influence on the measurement results. Besides the influence of different pathologies on posture, advancing age could have also negatively affected the postural control [52].

(c) The following discussion of the LIME effects focuses on the results for the spinal fusion data due to the best classification performance. The reason is that, on the one hand, local LIME effects may indicate characteristics of a healthy or pathologic subject for the respective vertebras and, on the other hand, it is also possible that the model learned a wrong relationship between the feature value and the class membership. For the identification of subject-specific characteristics, it is, therefore, important to reduce model mistakes.

Looking at the LIME values of the OCSVM and the binary RF approaches with the highest effects, showed that partly congruent results were present for the features with the highest effects for both approaches. However, there were also diverging results; for example, the pelvis more often had a higher relevance for the OCSVM. In general, only a little research addresses the agreement of XAI results for different classifiers, different XAI approaches [32], and different extracted features [31] in the context of biomechanical data and, consequently, more research is necessary. The exemplary LIME results for each of the presented subjects often showed effects for both classes (healthy and outlier). Therefore, the present study´s patients also showed spinal characteristics, which seemed to be similar to those of the healthy subjects.

Regarding the partly different/instable XAI results for both approaches, the aggregation of the results of different XAI approaches and different models on the same data might be an interesting approach to increase the robustness of XAI interpretations. In the context of feature selection, the ensemble method has already proven useful in generating more robust results [53,54]. The use of similar methods in the context of XAI could, thus, be useful and could help to increase the stability and, consequently, the trust in the XAI interpretations. The inclusion of global interpretations, e.g., through permutation importance or partial dependence plots [55], may be complementary and add more insights into the data. For a practical use in clinical settings, the appropriateness of an exclusive focus on LIME appears questionable. Therefore, the combination of different approaches seems necessary.

Looking at the LIME effects, the boxplots showed little variation of the LIME values between the 10 measurements of each subject most of the time. Therefore, in general, the same effects were identified for the measurements of each subject, which also speaks for the quality of the interpretation. These results can also be justified by the fact that previous works showed a high accordance between multiple static and dynamic measurements of the same subjects [38].

(d) The LIME values did not always show high effects for the vertebras related to the direct location of the spinal fusion. A possible reason for this might be that spinal fusion causes a higher pathologic deviation in other regions of the spine. This could also be explained by the connection instability that often develops after surgery [56,57]. However, more distant vertebral bodies also showed large effects, and they are usually in the regions of reverse curvature. They appeared to deviate from the physiological positions in the sense of maintaining balance to compensate for the altered statics of the fused vertebral bodies.

(e) Looking at the exemplarily results for the correctly classified subject 3459598 showed that the LIME values with the highest effect (OCSVM: Flexion extensions T4, T5, and L1; RF: Flexion extensions T2, T3, and T4) indicated an effect toward an outlier. The effects were similar for both approaches. In line with the above-mentioned aspects, the features with the highest effects did not directly map the vertebras where the spinal fusion was located (T10–L2). For the misclassified subject 8232865, the LIME values (OCSVM: Flexion extensions T3, T4, and T5; RF: Flexion extensions T2, T3, and T4; location of the spinal fusion: T1–L1) indicated an effect for the class of healthy subjects. However, the subject belonged to the class of spinal fusion. The misclassified subject showed very different patterns compared to the other subjects of the spinal fusion group. In addition to the still obvious vertebral body position data deviating from physiological posture, the very long fusion (long-axis fusions with more than four vertebral bodies are more common in scoliosis patients) also suggested that subject 8232865 suffered from severe scoliosis. In general, the mean values for the flexion extensions T3, T4, and T5 of the group of spinal fusion were above the mean values for the healthy. For the misclassified sample, this was the other way around. This might be the reason why the binary classifier failed to correctly classify the subject, because it learned an oversimplified relationship of the feature values to the class membership. The training data for the respective pathology should, therefore, be expanded, especially for subjects that show similar characteristics to the misclassified subject, so that the model is able to map the respective characteristics during the training phase. Further feature engineering through an automated feature extraction [37] or the inclusion of global spinal parameters (e.g., lordosis and kyphosis angle) might also be promising. For the OCSVM, a further possible reason for misclassification was that hyperparameters were not optimally chosen due to the hyperparameter search, because it was only based on the validation set performance related to the healthy subjects. Hyperparameter searching using a scoring metric that captures the classification results of healthy and patient data might be a promising alternative approach for improving performance.

Overall, the results highlighted the usefulness of the proposed XAI approach for explaining the predictions of the pathology-independent classifier. With the proposed XAI approach, it was possible to understand why subjects were classified (including why they were misclassified) and to reduce the black box character of the machine learning model. Therefore, the current study formed an important step for making OCSVM classifiers more applicable in clinical contexts.

Common inference-based statistical analysis methods often aim to find global effects between different groups of subjects. However, global interpretations are misleading in the case of highly individual subject characteristics, which are potentially relevant for class memberships. For spinal data, a previous work showed that highly individual patterns are present, which even enables the recognition of subjects [38]. For example, for the present groups of pathologies, the location of the spinal fusion was highly individual. Therefore, local instead of global interpretations gained high relevance. This highlights the clinical importance of the use of XAI tools, such as in the present study, for obtaining local interpretations.

Overall, the present results showed a high potential for the proposed pathology-independent classifier and no clear superiority of commonly used binary classification approaches. Limitations are to be mentioned in connection with the validity of the measurements while standing. Although a meta-analysis was able to confirm an overall reliable and valid measurement method for the assessment of spinal posture, this was particularly the case for global parameters such as thoracic kyphosis or lumbar lordosis. Pelvic parameters, such as obliquity or torsion, were less reliable and showed higher reliability in scoliosis patients than in healthy individuals, but one reason for this may be the smaller sample size [58]. However, further research has also shown that similar vertebral body deviations in the transverse plane of healthy persons could be measured with the help of rasterstereography [39], as another research group was able to perform with the help of CT and MRI images [59,60].

The sample of healthy subjects for training the pathology-independent classifier was relatively small. The discovered differences could, therefore, also be due to the sample and not due to actual differences in the subjects with pathologies. An expansion of the sample is, therefore, necessary for future studies. There were limitations regarding the sample of subjects and the large age difference between the groups. At this point, it cannot be excluded that the corresponding effects influenced the classification task. In following studies, the analysis should be repeated with matched groups of subjects. Further, the present study evaluated the posture while standing. Other positions (e.g., sitting), as demonstrated in [61], may influence spinal parameters and should also be considered in future works.

Interacting features might influence the dependency between the feature and, thus, the LIME values. Hence, possible interactions should be considered and analyzed in future works. For different contexts, different interpretation levels can be provided through selecting a maximum number of features to be displayed by LIME. However, there is no ground truth for the evaluation of the interpretation results. It is, therefore, difficult to evaluate if they were meaningful and appropriately mapped posture characteristics. Furthermore, according to the current state of research, there were no objective criteria to evaluate interpretability [55]. For the evaluation of the interpretation results, task performance might be a useful approach [62]. A comparison of clinical expert-based decisions with the XAI results might also be relevant for the additional evaluation of automated XAI interpretations.

The present study used static spinal data for the application of the methods. However, in the context of biologic gender classification, for example, dynamic data have proven superior for classification compared to the use of static data [37]. Furthermore, there are biomechanical reasons why dynamic data might better map pathologic differences [63,64]. Previous studies have shown that chronic back pain patients, in addition to altered thoracic–pelvic or lumbar–pelvic coordination in the transverse and frontal planes, also had different muscular control of the back muscles than healthy subjects [63,64]. In the transverse plane, coordination was less variable and more rigid, and the pelvis, lumbar spine, and thorax moved in phase; meanwhile, in the frontal plane, all three body parts showed looser and more variable coordination, especially at higher walking speeds, while rotational amplitudes were not affected [64]. The ipsilateral back muscles (on the side of the pain) showed increased muscle activity during the actual rather inactive swing leg phase and decreased activation during the double-supported stance phase [63,64]. A dynamic adaptation in gait to existing muscular or capsular deficits can also be assumed in osteoarthritis patients. These altered biomechanical parameters probably only become apparent through an asymmetrical and less-coordinated movement sequence. This is why the application of dynamic spinal data seems to have the potential to improve classification performance and should be evaluated in future works. In addition, studies have reported improved classification performance for feature extraction or mapping objects into an embedded space through learning a representation function (metric learning) [65]. Consequently, feature extraction and metric learning approaches should also be considered for future works. Regarding the used outlier detection algorithm, various other unsupervised approaches (e.g., isolation forest algorithm [40]), as well as semi-supervised outlier detection approaches [66], are proposed in literature. Regarding the used XAI algorithm for explaining the predictions, various other approaches have been reported (e.g., SHAP [34] and DeepLIFT [35]). As a next step, the comparison of the proposed approach with other algorithms and other XAI tools seems to be important.

As a possible field of application, except of the use in the clinical analysis of spinal data, the proposed approach might also be interesting in the field of ergonomics. Trunk posture classifiers are an emerging application for estimating spinal loads during manual lifting tasks that may lead to low back pain [67]. Many of the most-used and simple biomechanical models applied for estimating spinal loads utilize only a few parameters related to the trunk posture for estimating spinal loads [68]. The present study’s pathology-independent approach could possibly be expanded to automatically identify incorrect positions in real time by only learning the patterns of correct ergonomic positions.

## 5. Conclusions

The results suggested the potential suitability of the proposed pathology-independent approach. In the present study, no clear superiority of the commonly used binary classifiers compared to the proposed approach could be demonstrated. Static data did not seem to be optimally discriminant for mapping differences between the currently studied patient groups (especially for the subjects with back pain) and the healthy subjects. As a next step, spinal movement data should be used for classification to check if the dynamic data better map group differences and lead to increased classification performance. Moreover, metric learning approaches should be evaluated.

The proposed pathology-independent data-driven approach could be helpful for providing clinicians and therapists an objective orientation and to individually adapt and monitor therapy measures pre- and post-operatively. Overall, the approach might be beneficial for finding and addressing individual spinal characteristics. In the context of personalized medicine, the most relevant characteristics for each subject classification might be useful as an objective orientation and for an individual adaptation of therapy measures.

## Figures and Tables

**Table 1:** Subject characteristics and related trials.

	Subjects (*n*)	Male (*n*); Female (*n*)	Age (SD)	Hight cm (SD)	BMI (SD)	Further Information
Healthy ^1^ (asymptomatic)	25	12; 13	34.68 (12.07)	176.28 (8.83)	24.01 (3.45)	Repeated measurements at three points in time; walking without walking aids and pain; no acute or chronic diseases; no pregnancy; BMI < 30; WHO register (INT: DRKS00014325)
Back pain	32	14; 18	44.53 (14.84)	174.00 (11.00)	26.01 (4.79)	Area of pain: 6% thoracic spine (TS), 72% lumbar spine (LS), and 22% TS + LS; no acute fractures, walking aids, or acute/chronic illnesses that prevent safe walking; WHO register (INT: DRKS00013145)
Spinal fusion	34	20; 14	56.26 (15.40)	171.00 (11.00)	26.95 (4.43)	Spinal fusion somewhere between C7 and L5; no acute fractures, walking aids, or acute/chronic illnesses that prevent safe walking; WHO register (INT: DRKS00013145)
Osteoarthritis ^2^	60	29; 31	64.00 (11.27)	171.00 (9.15)	25.68 (2.35)	30 knee osteoarthritis and 30 hip osteoarthritis; walking without walking aids; no walking impairments that prevent safe walking; no acute or chronic diseases; no pelvic or spinal surgery; no pregnancy; BMI < 30; WHO register (INT: DRKS00017240)

^1^ The dataset is part of the dissertation project of Friederike Werthmann. ^2^ The dataset is part of the dissertation project of Claudia Wolf.

**Table 2:** Classification results (mean and standard deviation) for the pathology-independent one-class classifier (OCSVM) and the binary classification approach using a random forest classifier (RF). Absolute class predictions were conducted according to the calculated/calibrated prediction probabilities. MCC, Matthews correlation coefficient; F1, F1 score; CM, confusion matrix.

		One Class SVM					Binary RF Classifier				
	Data	F1	MCC	BSS	CM		F1	MCC	BSS	CM	
Synthetic	S1	0.96 ± 0.05	0.92 ± 0.1	0.84 ± 0.12	220	2	1.0 ± 0.0	1.0 ± 0.01	0.93 ± 0.02	239	0
					20	238				1	240
	S2	0.99 ± 0.01	0.99 ± 0.02	0.95 ± 0.02	237	0	0.98 ± 0.03	0.96 ± 0.07	0.88 ± 0.05	230	1
					3	240				10	239
	S3	0.89 ± 0.03	0.77 ± 0.05	0.61 ± 0.12	203	19	0.95 ± 0.04	0.90 ± 0.09	0.79 ± 0.12	223	6
					37	221				17	234
	S4	0.82 ± 0.09	0.65 ± 0.19	0.46 ± 0.23	192	38	0.90 ± 0.04	0.82 ± 0.06	0.65 ± 0.09	217	23
					48	202				23	217
Real	BP	0.54 ± 0.13	0.13 ± 0.19	0.02 ± 0.10	149	165	0.62 ± 0.17	0.08 ± 0.34	–0.08 ± 0.35	98	113
					91	155				142	207
	Spinal fusion	0.80 ± 0.12	0.57 ± 0.23	0.33 ± 0.28	194	78	0.74 ± 0.25	0.45 ± 0.25	0.36 ± 0.31	171	86
					46	262				69	254
	Osteoarthritis	0.69 ± 0.04	0.21 ± 0.12	0.35 ± 0.30	138	230	0.78 ± 0.09	0.19 ± 0.21	41.28 ± 0.35	73	107
					102	370				167	493

**Table 3:** Results for the spinal fusion subjects (mean and standard deviation of the 10 measurements per subject), as well as localization of the spinal fusion and the features with the highest relevance according to the LIME results. The probabilities are for classification as an outlier/patient. P, pelvis.

Subject ID	OCSVM Mean Prediction Probability	RF Mean Prediction Probability	Location of Spinal Fusion	LIME OCSVM	LIME RF
1962247	1.00 ± 0.00	0.92 ± 0.00	L1–S1	T8, T9, P	T9, T10, T7
3459598	1.00 ± 0.00	0.93 ± 0.00	T10–L2	T4, L1, T5	T3, T4, T2
7741511	0.99 ± 0.00	0.90 ± 0.01	L5–S1	T9, P, T3	T9, T10, T7
5777016	0.98 ± 0.01	0.92 ± 0.00	T10–L3	L4, P, P	T9, T7, T5
7475130	0.96 ± 0.01	0.73 ± 0.01	L3–S1	T4, T5, T6	T4, T3, T2
9342653	0.96 ± 0.01	0.80 ± 0.04	L4–L5	L4, P, L1	T9, T7, T8
3729138	0.90 ± 0.01	0.87 ± 0.02	L2–L3	P, P, T8	T3, T4, T7
5536002	0.87 ± 0.02	0.71 ± 0.03	T6–L3	L4, P, T2	T3, T4, T7
6705867	0.87 ± 0.02	0.84 ± 0.01	L5–S1	P, T12, L4	T3, T4, T12
5247355	0.83 ± 0.03	0.88 ± 0.02	L5–S1	P, L4, T8	T4, T5, T8
5297873	0.81 ± 0.03	0.80 ± 0.02	L4–L5	P, P, T7	T8, T7, T5
5408449	0.80 ± 0.02	0.71 ± 0.02	T4–L1	T7, T8, T12	T5, T4, L1
3336746	0.78 ± 0.02	0.93 ± 0.01	L3–L5	P, T8, P	T4, T5, T8
9747703	0.77 ± 0.01	0.94 ± 0.01	L3–L5	P, L4, T3	T4, T5, T8
2324908	0.76 ± 0.06	0.90 ± 0.01	T11–L3	P, L4, L3	T3, T4, T7
8398276	0.75 ± 0.05	0.43 ± 0.03	T10–L2	L4, P, T11	T3, T4, T7
3012624	0.70 ± 0.07	0.83 ± 0.07	L4–L5	P, T8, T12	T3, T4, T7
7767875	0.62 ± 0.02	0.82 ± 0.02	C6–T2	P, P, L4	T5, T4, T6
5815929	0.60 ± 0.03	0.92 ± 0.00	T12–L2	P, L4, P	T4, T5, T8
9621669	0.56 ± 0.09	0.34 ± 0.05	T12–L2	T4, T3, T5	T3, T4, T2
1082776	0.55 ± 0.00	0.43 ± 0.05	L2–L4	L4, T4, T5	T3, T11, L4
649887	0.53 ± 0.01	0.82 ± 0.01	L4–L5	VP, T3, T9	T3, T3, T4
7550216	0.53 ± 0.00	0.82 ± 0.02	T10–L5	VP, T3, T9	T3, T9, T6
3943929	0.51 ± 0.00	0.88 ± 0.01	L3–L4	L4, T9, L3	T9, T3, T4
5584179	0.51 ± 0.01	0.89 ± 0.01	L4–L5	T9, T4, VP	T9, T3, T8
6777530	0.51 ± 0.01	0.86 ± 0.01	L2–L4	VP, T3, L3	T3, T9, T6
9299446	0.50 ± 0.01	0.32 ± 0.03	T5–T10	L3, VP, T12	T3, T7, T3
632814	0.45 ± 0.01	0.76 ± 0.06	L4–S1	L4, VP, T3	T3, T9, T6
3035442	0.42 ± 0.00	0.71 ± 0.02	T11–L2	L4, VP, T3	T3, T9, T8
6683738	0.41 ± 0.09	0.11 ± 0.01	L4–L5	T3, L1, T2	T3, T2, T10
2064644	0.31 ± 0.06	0.19 ± 0.05	L4–S1	P, T7, T11	T10, T9, T7
9664225	0.08 ± 0.04	0.44 ± 0.13	T6–T11	T12, L4, T11	T3, T4, T12
1084868	0.03 ± 0.00	0.08 ± 0.01	T6–T10	T8, T9, P	T9, T10, T8
8232865	0.00 ± 0.00	0.37 ± 0.02	T1–L1	T4, T3, T5	T3, T4, T2

## Data Availability

The data are not publicly available due to restrictions on the use of clinical patient data.

## Appendix A

**Table A1:** Variables used for modeling. Variables were measured in sagittal, frontal, and transversal plane. The descriptions were adapted from the DIERS manual.

Variable	Plane	Description
VP/C7 (Vertebra prominens/7. cervical vertebral body) T1–T12 (Thoracic spine) L1–L4 (Lumbar spine)	Sagittal: Vertebral sagittal Flexion and Extension (°)	The parameter describes the inclination of the calculated vertebra in space (relative to a plumb/gravity line) as seen from a left view. The angle (in degrees) is calculated from the projection of the vertebra in a sagittal plane (rotation and lateral flexion are ignored). A positive value means a forward tilt of the vertebra (flexion).
	Frontal: Vertebral Lateral Flexion (°)	The parameter describes the lateral inclination of the vertebra in space (relative to a plumb/gravity line) as seen from a posterior–anterior view. The angle (in degrees) is calculated from the projection of the vertebra in the coronal plane (rotation and sagittal extension/flexion are ignored). A positive value means a tilt of the vertebra to the left (lateral flexion left).
	Transversal: Vertebral Rotation (°)	The vertebral rotation describes the rotation of a vertebra in the transversal plane (relative to the neutral pelvis). A positive value means a vertebra is rotated to the left (counterclockwise) when seen from behind. The rotation of vertebral bodies happens in situ and, therefore, the direction of rotation between the surface and vertebral rotation changes. Hence, a surface rotation to the right, mathematically represented with a +, becomes a vertebral body rotation to the left. This is due to the calculation process in which a vector is used that points from the Processus spinosus towards the middle of the vertebral body, meaning that the surface rotation changes its direction within the vertebral body.
Pelvis	Pelvic Obliquity (°)	A line is drawn from DL to DR (left and right dimple) and is compared to a horizontal line representing the horizon. The angle (in degrees) between them is measured. A positive value means that the right pelvis is elevated.
	Pelvic Torsion (dimples) (°)	The parameter describes the torsion of the surface normals on the two lumbar dimples.
	Pelvic Inclination (dimples) (°)	The parameter describes the mean vertical torsion of the two surface normals on DL and DR.
	Pelvic Inclination (symmetry line) (°)	The parameter describes the angle of the vertical components of the surface normals on point DM (dimple midpoint) based on the horizontal.
	Pelvic Rotation (°)	The pelvic rotation is the rotation in the transversal plane of the right dimple relative to a reference coronal plane that is defined from the system setup, perpendicular to the camera-projection axis. A positive value means the pelvis is rotated to the left when seen from behind (the value is corrected * (−1)).
